# Clofarabine/busulfan-based reduced intensity conditioning regimens provides very good survivals in acute myeloid leukemia patients in complete remission at transplant: a retrospective study on behalf of the SFGM-TC

**DOI:** 10.18632/oncotarget.26391

**Published:** 2018-11-27

**Authors:** Amandine Le Bourgeois, Myriam Labopin, Mathieu Leclerc, Régis Peffault de Latour, Jean-Henri Bourhis, Patrice Ceballos, Corentin Orvain, Hélène Labussière Wallet, Karin Bilger, Didier Blaise, Marie-Thérese Rubio, Thierry Guillaume, Mohamad Mohty, Patrice Chevallier, on behalf of Société Francophone de Greffe de Moelle et de Thérapie Cellulaire

**Affiliations:** ^1^ Department of Hematology, CHU Hôtel Dieu, Nantes, France; ^2^ Department of Hematology, Hôpital Saint Antoine, Paris, France; ^3^ Department of Hematology, Hôpital Henri Mondor, Créteil, France; ^4^ Department of Hematology, Hôpital Saint Louis, Université Paris 7, Denis Diderot, Paris, France; ^5^ Department of Hematology, Hôpital Gustave Roussy, Paris, France; ^6^ Department of Hematology, CHU de Montpellier, Montpellier, France; ^7^ Department of Hematology, CHU d’Angers, Angers, France; ^8^ Department of Hematology, Centre Hospitalier Lyon Sud, Lyon, France; ^9^ Department of Hematology, CHU Strasbourg, Strasbourg, France; ^10^ Department of Hematology, Centre de Recherche en Cancérologie de Marseille, Institut Paoli Calmettes, Marseille, France; ^11^ Department of Hematology, CHU Nancy, Nancy, France

**Keywords:** allogeneic stem cell transplantation, clofarabine, busulfan, reduced intensity conditioning regimen, acute myeloid leukemia

## Abstract

**Background:**

Clofarabine has been proved to have higher anti-leukemic myeloid activity compared to fludarabine, a drug extensively used as part of reduced intensity conditioning (RIC) for allogeneic stem cell transplantation (allo-SCT).

**Results:**

Eighty-four patients were included. The majority of patients had acute myeloid leukemia (AML, *n* = 63). Sixty-one patients were in complete remission (AML *n* = 55). With a median follow up of 31 months (range: 5.7–74.1), 2-year overall (OS) and disease-free (DFS) survivals, relapse incidence (RI), non-relapse mortality (NRM) and graft-versus-host disease (GVHD)/relapse free survival (GRFS) were 64.5% (53.8–75.2); 57.2% (46.2–68.2); 27.7% (18.2–37.9); 15.1% (8.2–23.9) and 43.6% (32.5–54.7), respectively. Considering AML in remission, 2-year OS, DFS, RI, NRM and GRFS were 74.2% (62–86.5); 66.8% (53.6–79.9); 23.4% (12.7–36); 9.8% (3.5–19.9) and 50.9% (36.9–64.9), respectively. Two-year outcomes were similar between CloB2A1 and CloB2A2 sub-groups. In multivariate analysis, active disease at transplant was the only factor adversely impacting 2 years outcomes.

**Conclusions:**

CloB2A2/A1 RIC regimen provides very good results for AML patients allografted in CR and could be retained as a new RIC platform for these patients.

**Materials and Methods:**

This was a retrospective study including all patients who received a clofarabine/busulfan based RIC allo-SCT for myeloid malignancies and reported within the SFGM-TC registry. RIC regimen consisted of clofarabine 30 mg/m^2^/day 4 to 5 days (Clo), busulfan 3.2 mg/kg/day 2 days (B2) and 2.5 mg/kg/day of rabbit anti-thymocyte globulin 1 or 2 days (A1 or A2). The primary objective of the study was to report the main outcomes of the whole cohort at 2 years.

## INTRODUCTION

Reduced-intensity conditioning regimens (RIC) were successfully introduced twenty years ago to decrease the toxicity of myeloablative allogeneic stem cell transplantation (allo-SCT). This resulted in the doubling of the number of allo-SCT performed each year, because older patients and those with comorbidities could tolerate such a procedure [1–7]. The FB2A2 regimen (fludarabine 30 mg/kg/day for 5 days, busulfan 3.2 mg/kg/d iv for 2 days, and rabbit anti-thymocyte globulin (ATG) 2.5 mg/kg/d for 2 days) was rapidly established as one of the standards of care for RIC regimen in Europe, especially in France [4–9].

As relapse remains as one of the main causes of morbidity and mortality after allo-SCT, strategies to improve outcomes of patients were developed thereafter, including modification of the conditioning regimen using the same drugs at higher dose (reduced-toxicity myeloablative conditioning regimen, FB3/FB4) [10–12] or other drugs with higher anti-leukemic activity. For the latter, clofarabine, a second-generation purine analogue, has been proven to have both anti-myeloid [13–15] and anti-lymphoid activity [16, 17]. Recently, clofarabine (Clo) has been used as part of fludarabine-busulfan-based conditioning regimen instead of fludarabine, showing encouraging results. Thus, the CloB2A2 RIC regimen (clofarabine 30 mg/kg/day for 5 days, busulfan 3.2 mg/kg/day iv for 2 days, ATG 2.5 mg/kg/day for 2 days) has been validated in a prospective phase II study including 14 patients with acute lymphoblastic leukemia (ALL), 11 with acute myeloid leukemia (AML) and 5 with myelodysplastic syndrome (MDS). At one year, the overall survival (OS) and leukemia free survival (LFS) were of 63% and 57%, respectively, and the non-relapse mortality (NRM) only of 3.3%, suggesting a very good safety profile of the RIC regimen [18]. More intensive clofarabine/busulfan RIC regimens (CloB4) have also shown good safety profile after transplant for ALL [19]. Our group retrospectively compared the CloB2A2 (*n* = 39) versus FB2A2 (*n* = 316) RIC regimens in a cohort of patients with myeloid disease, and showed a significant OS benefit for the former in multivariate analysis [20].

The aim of the present study was to confirm the favorable impact of the CloB2A2 RIC regimen in a larger cohort of patients with myeloid malignancies. Also, because some centers decreased the dose of ATG (CloB2A1) after 2014 with the aim to decrease relapse, [4] we also assessed the effect of this lower dose on outcomes.

## RESULTS

### Characteristics of patients (Table 1)

**Table 1:** Patients’ characteristics

	Whole cohort *N* = 84	CloB2A1 *n* = 41	CloB2A2 *n* = 43	*p*-value
Median follow-up: months (range)	30.9 (5.7–74.1)	20.7 (5.7–34.3)	38.9 (20.5–74.1)	
Median year of transplant	2014 (09–16)	2015 (14–16)	2013 (09–16)	<0.001
Gender: males	47 (56%)	25 (61%)	22 (51%)	0.36
Median age at transplant: years (range)	61.6 (20.6–73.9)	65 (24.6–73.9)	60.8 (20.6–71.1)	0.028
Diagnosis: AML MDS/MPS	63 (75%) 21 (25%)	31 (75.6%) 10 (24.4%)	32 (74.4%) 11 (25.6%)	0.9
Status at transplant: CR1 CR2 Active disease	49 (58.3%) 12 (14.3%) 23 (27.4%)	27 (65.8%) 5 (12.2%) 9 (22%)	22 (51.2%) 7 (16.3%) 14 (32.5%)	0.39
Recipient CMV status: +	40 (48%)	23 (56%)	17 (39.5%)	0.12
Type of donor: Gender: males Sibling MUD CMV status: +	52 (62%° 34 (40.5%) 50 (59.5%) 24 (29%)	27 (66%) 19 (46.3%) 22 (53.7%) 9 (22%)	25 (58%) 15 (34.9%) 28 (65.1%) 15 (35%)	0.46 0.285 0.19
Sex matching Female → Male Others	13 (15.5%) 71 (84.5%)	5 (12.2%) 36 (87.8%)	8 (18.6%) 35 (81.4%)	0.47
Stem-cell source Bone Marrow PBSC	1 (1.2%) 83 (98.8%)	0 (0%) 41 (100%)	1 (2.3%) 42 (97.7%)	0.33
Previous transplant: No Previous allo-SCT Previous auto-SCT	75 (89.3%) 7 (8.3%) 2 (2.4%)	39 (95.1%) 2 (4.9%) 0 (0%)	36 (83.7%) 5 (11.6%) 2 (4.7%)	0.19
GVHD prophylaxis: -Csa -Csa + MTX -Csa + MMF	44 (52.4%) 3 (3.6%) 37 (44%)	18 (43.9%) 0 (0%) 23 (56.1%)	26 (60.5%) 3 (7%) 14 (32.5%)	0.04
Dose of clofarabine 120 mg/m^2^ ≥150 mg/m^2^	23 (27.3%) 61 (72.7%)	1 (2.5%) 40 (97.5%)	22 (51.2%) 21 (48.8%)	<0.001

Abbreviations: AML: acute myeloid leukemia, MDS: myelodysplastic syndrome; MPS: myeloproliferative syndrome; CR: complete remission; BM: bone marrow, MUD: matched unrelated donor; CMV: cytomegalovirus; PBSC: peripheral blood stem cells; Allo-SCT: allogeneic stem cell transplant, Auto-SCT: autologous stem cell transplant; Csa: cyclosorine, MTX: methotrexate; MMF: mycophenolate mofetil.

Between January 2008 and December 2016, 84 patients (males *n* = 47, median age: 61.6 years) met the inclusion criteria. The outcomes of some patients have already been reported [16, 18]. The majority of patients had AML (*n* = 63), including 43 cases in CR1, 12 in CR2 and 8 with active disease at transplant. According to ELN-risk classification [21], 7 AML cases were classified as favorable, 24 as intermediate-1, 8 as intermediate-2 and 6 as unfavorable (unknown *n* = 18). There were 21 patients with MDS (*n* = 18) or MPS (*n* = 3), including 6 in CR1 (all MDS) and 15 with active disease at transplant. All patients but one (bone marrow graft) received peripheral blood stem cell as graft source. All patients received cells from matched donors (sibling *n* = 34; unrelated *n* = 50).

There were no significant differences in terms of characteristics between CloB2A2 (*n* = 43, median follow-up 39 months) and CloB2A1 (*n* = 41, median follow-up 21 months) patients, except for the median age of the cohort (65 vs 61 years, *p* = 0.02), the dose of clofarabine (≥150 mg/m2: 97.5% vs 49% of the patients, *p* < 0.001), which were higher in the latter group and, as expected, the median year of transplant (2013 vs 2015, *p* < 0.001) (Table 1). All patients engrafted except one CLOB2A1 case.

### Outcomes

With a median follow up of 31 months (range: 5.7–74.1) for patients alive, the 2-year OS, LFS, RI, and NRM were 64.5% (53.8–75.2), 57.2% (46.2–68.2), 27.7% (18.2–37.9) and 15.1% (8.2–23.9), respectively. The CI of 100-day acute GVHD grade II-IV and III-IV were 20.2% (12.4–29.4) and 7.1% (2.9–14), respectively. The 2-year CI of extensive chronic GVHD was 9.6% (4.1–17.8%). The 2-year GRFS was 43.6% (32.5–54.7). The median time to relapse/progression from the transplant was 5.6 months (range: 0.59–31.5). At last follow-up, 32 patients had died, mainly due to relapse (*n* = 17), then to GVHD (*n* = 6), infection (*n* = 6), hemorrhage *n* = 1; microangiopathy *n* = 1, and non-identified transplant-related *n* = 1.

**Figure 1:**
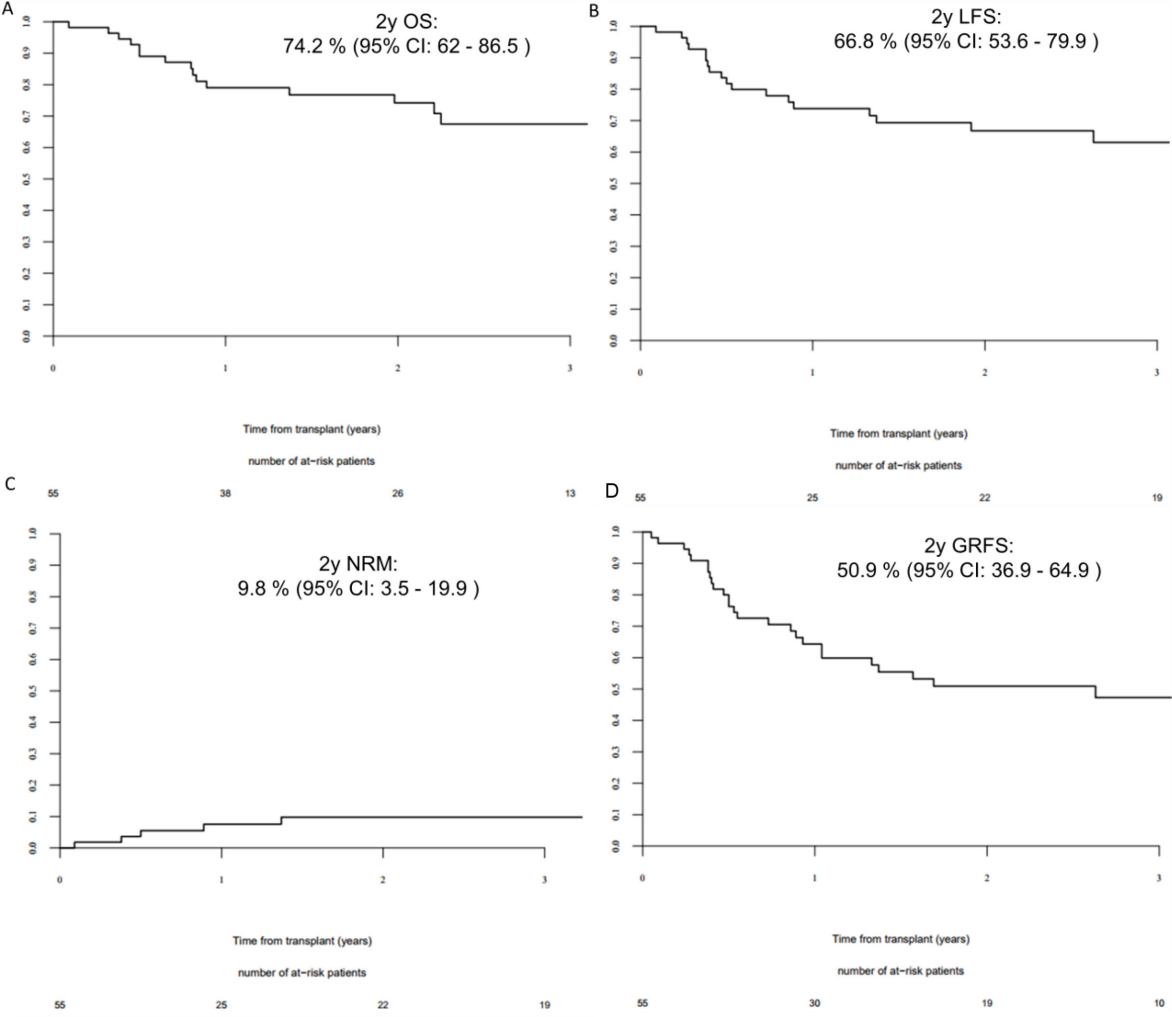
2 year outcomes for AML patients in complete remission at transplant (**A**) 2-year overall survival for acute myeloid leukemia (AML) patient in complete remission (CR); (**B**) 2-year leukemia free survival for AML patients in CR; (**C**) 2-year non-relapse mortality for AML patients in CR; (**D**) 2-year GVHD-free/relapse-free survival for AML patient in CR.

Considering AML in CR (*n* = 55), the 2-year OS, LFS, RI, NRM and GRFS were 74.2% (62–86.5); 66.8% (53.6–79.9); 23.4% (12.7–36); 9.8% (3.5–19.9) and 50.9% (36.9–64.9) respectively. The 100 day grade 2–4 and 3–4 acute GVHD CI were 16.4 % (95% CI: 8–27.3) and 1.8% (95% CI: 0.1–8.6), respectively, while the 2-year CI of extensive chronic GVHD was 10.4 % (95% CI: 3.7–21.2) (Figure 1).

### Univariate analysis

In univariate analysis, active disease at transplant was associated with lower 2-year survivals (OS: 37.7% [17.3–58.1] vs CR1: 75.5% [62.8–88.2] vs CR2: 75% [50.5–99.5], *p* = 0.001; LFS: 31.8% [12.4–51.3] vs CR1: 69.9% [56.5–83.2] vs CR2: 58.3% [30.4–86.2], *p* = 0.0004; GRFS: 19.6% [2.1–37] vs CR1: 53.9% [39.1–68.7] vs CR2: 50% [21.7–78.3], *p* = 0.0006) and higher incidence of 2-year NRM (31.8% [13.6–51.8] vs CR1: 9% [2.8–19.8] vs CR2: 8.3% [0.4–32.4], *p* = 0.008). Also, older age (>median) was associated with better GRFS (56.3% [40.3–72.3] vs 32.1% [17.8–46.4], *p* = 0.03), while transplant from a MUD donor was associated with higher acute grade 2–4 and 3–4 GVHD (28% [16.3–40.9] vs 8.8% [2.2–21.3], *p* = 0.04; and 12% [4.8–22.7] vs 0%, *p* = 0.03).

**Figure 2:**
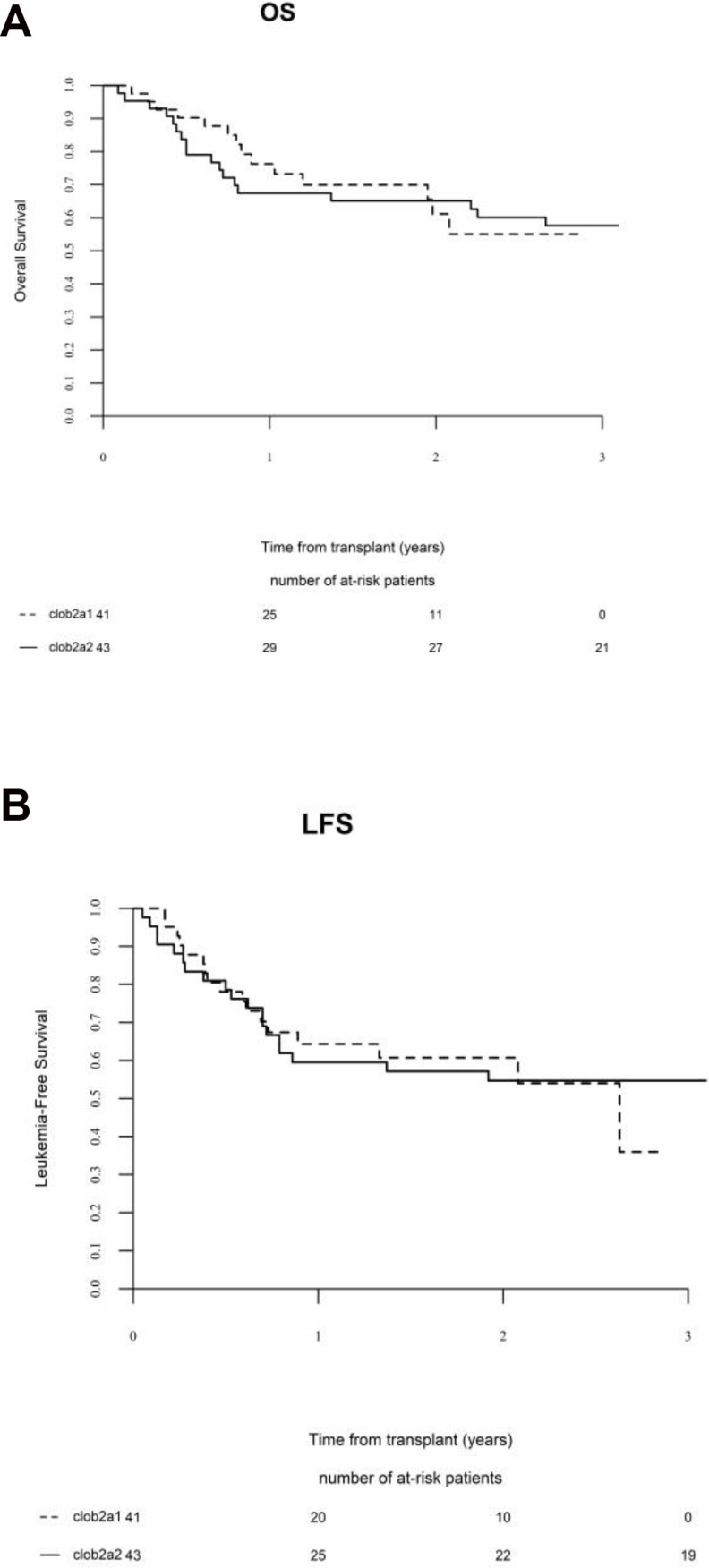
Comparison of (**A**) overall survival (OS) and (**B**) leukemia-free survival (LFS) between CloB2A1 and CloB2A2 sub-groups.

Finally, there were no differences in terms of 2-year outcomes between CloB2A1 and CloB2A2 subgroups considering the whole cohort (Table 2 and Figure 2) or only AML patients in CR (Table 2).

**Table 2:** Comparison of 2-year outcomes between CloB2A1 and CloB2A2 sub-groups

		Relapse incidence	NRM	LFS	OS	GRFS	CI of 100-day grade II–IV acute GVHD	CI of 100-day grade III-IV acute GVHD	CI of extensive chronic GVHD
**All patients**	**CloB2A1**	28.8% [15.1–44]	10.5% [3.2–22.7]	60.8% [44.9–76.6]	61.2% [43.8–78.6]	45% [27.9–62]	22% [10.7–35.7]	7.3% [1.9–18]	6.1% [1–18.2]
	**CloB2A2**	26.3% [14–40.3]	19% [8.8–32.2]	54.7% [39.6–69.7]	65.1% [50.9–79.4]	41.7% [27–56.5]	18.6% [8.6–31.5]	7% [1.8–17.2]	12% [4.3–24.1]
	***p*-value**	0.51	0.55	0.93	0.94	0.97	0.44	0.99	0.43
**AML in RC**	**CloB2A1**	25.6% [10.8–43.6]	4.2% [0.3–18.1]	70.2% [52.4–88]	74.4% [55.7–93.1]	47.8% [27.2–68.4]	13.3% [4.1–28.1]	0%	8.6% [1.3–24.7]
	**CloB2A2**	20% [7–37.7]	16% [4.8–33]	64% [45.2–82.8]	72% [54.4–89.6]	52% [32.4–71.6]	20% [7.1–37.6]	4% [0.3–17.4]	12% [2.9–28.2]
	***p*-value**	0.30	0.14	0.97	0.50	0.86	0.95	0.27	0.72

Abbreviations: AML: acute myeloid leukemia, CR: complete remission, GRFS: GVHD-free/relapse-free survival, LFS: leukemia free survival, NRM: non-relapse mortality, OS: overall survival, CI: cumulative incidence.

### Multivariate analysis

In multivariate analysis, active disease at transplant was the only factor adversely impacting 2 year outcomes: OS: HR: 3.83 (95% CI: 1.77–8.29, *p* = 0.0006; DFS: 3.99; 95% CI: 1.90–8.35, *p* = 0.0002; GRFS: HR: 3.17; 95% CI: 1.65–6.08, *p* < 0.0001; relapse: HR: 2.60; 95% CI: 1.00– 6.77, *p* = 0.04; and NRM: HR: 7.02; 95% CI: 2.02–24.36, *p* = 0.002). Neither the conditioning regimen (CloB2A2 vs CloB2A1) nor the dose of clofarabine (120 mg/m^2^ vs ≥150 mg/m^2^) influenced the outcomes.

## DISCUSSION

Here, we have reported the largest retrospective cohort of patients allografted with a clofarabine/busulfan-based RIC regimen for myeloid malignancies. Although there are some biases common to all retrospective studies, very encouraging results have been observed with this platform, especially for AML cases in CR at transplant, as the 2-year OS was almost 75% while LFS and GRFS reached 67% and 51%, respectively. Overall, this compared very favorably with the results obtained with the standard of care RIC regimen used in France, the FB2A2 regimen [4–9]. This also confirmed our previous data for AML patients in CR where the 2-year OS and LFS for FB2A2 patients were only 38% and 38%, respectively, while it was 79.2% and 70.8% for the CloB2A2 group [20].

GRFS, a new composite end-point reflecting the quality of life of patients after transplant, has been poorly studied so far in the context of RIC setting. Here again, our results compared favorably with those published, as it is estimated that only approximately one-quarter of adult patients transplanted for malignant disease survived without at least 1 of the following major complications, grade 3–4 acute GVHD, chronic GVHD requiring systemic treatment, relapse, or death, during the first 12 months after HCT. Indeed, in the study by Holtan and colleagues, the first to consider this outcome after transplant, the estimated 1-year GRFS was only 26% after RIC [22]. A recent EBMT study, including more than 20000 cases of AML in CR1 or CR2, showed a GRFS of 40% at 3 years after both myeloablative and RIC regimens [23].

As recognized years ago for both myeloablative and RIC regimens [24], including fludarabine/busulfan based RIC regimens [8, 20], status at transplant remains the most important factor predicting survival for patients in our series. As such, all effort should be targeted to inducing CR before transplant, keeping in mind that CR1 and CR2 cases achieved similar survivals here. This highlights the potential impact of the depth of the response before the transplant, even in CR2. Measurable residual disease (MRD) for AML may have a decisive role in risk-stratification and prediction of outcomes after transplant to guide the intensity and the quality, in terms of anti-leukemic activity, of the conditioning regimen. For example, pre allo-SCT molecular MRD evaluation of NPM1 mutation was shown to be a powerful predictor of post allo-SCT outcomes in AML undergoing allo-SCT in cytologic CR [25]. Other data suggested the same for MRD evaluation of WT1 expression before transplant [26]. In cases with no cytogenetic or molecular markers associated with the AML, MRD evaluated by flow cytometry can also be an effective tool to predict relapse risk after transplant [27, 28]. As suggested before, maybe it is time to move towards a MRD-based definition of CR [29], especially now that the establishment of a consensus on measurement and application of MRD in AML has been achieved [30].

Compared to our previous report, one of the most original results here is the impact of decreasing the duration of ATG treatment to one day instead of two. It has been shown that reducing the dose of ATG, albeit possibly associated with less relapse [31], may be more damaging in terms of severe acute or chronic GVHD in the setting of fludarabine/busulfan-based RIC regimen [32, 33]. This seems not to be the same for clofarabine/busulfan-based RIC regimen as we observed similar survivals and GVHD incidences using CloB2A2 or CloB2A1, suggesting also that only one day of ATG is sufficient in this context.

As clofarabine seems to have a high anti-leukemic activity, its use for alternative transplant as part of the conditioning regimen may be also appropriate. Although scanty data exist for cord blood transplant [34], sequential approaches of debulking with clofarabine/cytosine arabinoside followed by the conditioning regimen have been recently published with encouraging results for refractory AML [35, 36]. We have also recently reported our experience of a “Clo-Baltimore” RIC regimen for myeloid malignancies where fludarabine is replaced by clofarabine as part of a standard Baltimore regimen. The results of this approach in 36 patients showed 18-month OS and DFS of 72% + 7.5%, and 63.8 + 8%, respectively, and a GRFS of 52.6 + 8% [37].

Finally, the question on how to improve our results by post-transplant strategies to avoid relapse is a real issue. FMS-like tyrosine kinase 3 inhibitors for FLT3-ITD-positive AML [38] or prophylactic azacitidine and donor lymphocyte infusions [39] after allo-SCT may become also a new standard of care in the future for both clofarabine/fludarabine-busulfan based RIC regimen allo-SCT.

In conclusion, CloB2A2/A1 RIC regimen provide very good results for AML patients allografted in CR and should be retained as a new RIC platform for these patients. Prospective studies are definitively needed to compare CloB2 vs FB2 RIC regimens.

## MATERIALS AND METHODS

### Eligibility criteria and study design

This was a retrospective study conducted on behalf of the Société Francophone de Greffe de Moelle et de Thérapie Cellulaire (SFGMTC), including all adults (≥18 years old) who received a clofarabine/busulfan based RIC allo-SCT for myeloid malignancies (AML, MDS, myeloproliferative syndrome (MPS)) and reported within the SFGM-TC registry. Data were obtained through PROMISE, an internet-based system shared by all European transplantation centers. All patients gave informed consent for collection of their personal data in this data base.

### Conditioning regimen

The RIC regimen consisted of clofarabine 30 mg/m^2^/day for 4 to 5 days (Clo), busulfan 3.2 mg/kg/day for 2 days (B2) and rabbit anti-thymocyte globulin (A) 2.5 mg/kg/day for 1 or 2 days (A1 or A2). CloB2A2 was initially used as the conditioning platform, while since 2014 some centers have used CloB2A1 in order to try to decrease relapse and infections, and thus improve on the results of the CloB2A2 patients [4].

### Statistical analyses

The clinical outcomes studied were overall survival (OS), leukemia-free survival (LFS), relapse incidence (RI) and non-relapse mortality (NRM). OS was defined as the time from day 0 of allo-SCT to death or last follow-up for survivors. LFS was defined as time from day 0 of allo-SCT to time without evidence of relapse or disease progression. Relapse was defined as any event related to re-occurrence of the disease. NRM was defined as death from any cause without previous relapse or progression. Probabilities of OS and LFS were calculated using the log-rank test and Kaplan–Meier graphical representation. Cumulative incidence functions (CIF) [40] were used to estimate RI and NRM in a competing risk setting. In order to study acute and chronic graft versus host disease (GVHD), we considered death and relapse as competing events. Acute and chronic GVHD were diagnosed and graded according to standard criteria [41, 42]. The GVHD-free/relapse-free survival (GRFS), defined as alive with no previous grade III-IV aGvHD, no extensive chronic GvHD and no relapse [22, 23], was also studied. Survival probabilities are presented as percentages and 95% confidence intervals (95% CI).

Univariate analyses were performed using the log rank test for OS and LFS and the Gray test for CIF. Characteristics considered for univariate analysis were the conditioning regimen (CloB2A2 vs CloB2A1), recipient gender (male vs female) and age (< or ≥ median, continuous); years of transplant (< or ≥ median), disease (AML vs MDS/MPS), disease status at transplant (first complete remission (CR1) vs second CR (CR2) vs active disease), type of donors (sibling vs matched unrelated donor (MUD)), donor gender (male vs female) and age (< or ≥ median), gender matching (female donor for male recipient vs others), recipient and donor CMV status (+ vs -), GVHD prophylaxis (cyclosporine vs cyclosporine + mycophenolate mofetil) and dose of clofarabine (120mg/m^2^ vs ≥150 mg/m^2^). Main outcomes were also compared between CloB2A2 vs CloB2A1 cases, considering only AML patients in CR.

Multivariate analyses were performed using the Cox proportional-hazard model. Factors significantly associated with the outcome in univariate analysis and factors known to influence outcomes were included in the multivariate analysis.

All tests were two-sided and *P* values < 0.05 were considered as indicating a statistically significant association. Analyses were performed using the R statistical software version 3.2.3 (available online at http://www.R-project.org).
